# FORMATIVE EVALUATION TRIAL OF THE “MY HEALTH PRIORITIES” PROGRAM IN SOUTHERN OLDER AFRICAN AMERICANS: THE BHIP STUDY

**DOI:** 10.1093/geroni/igad104.1422

**Published:** 2023-12-21

**Authors:** Deborah Ejem

**Affiliations:** University of Alabama at Birmingham, Birmingham, Alabama, United States

## Abstract

Older African Americans (AA) with multiple chronic conditions (MCCs) living in the Deep South are less likely to have access to early palliative care (PC) despite experiencing higher symptom burden and healthcare use, and poorer communication around goals of care. This disparity in PC use may partly be due to a lack of culturally-responsive care practices that effectively activate AAs with MCCs to identify their own values and priorities for end-of-life care. We conducted a formative evaluation of the web-based, self-directed “My Health Priorities” Identification Program to determine cultural acceptability and feasibility of use among AAs with MCCs in a primary care setting. We are now recruiting 20 AA patient with MCC and caregiver dyads from UAB Kirklin Primary Care Clinic. Recruited dyads will complete the “My Health Priorities” program and participate in semi-structured acceptability interviews. Patients and caregivers will also complete pre- and post-test measures of perception of care, treatment burden, shared decision-making, and communication exchange. Preliminary findings from six dyads suggest that the current program lacks spirituality-specific content. Dyads also expressed that the exemplar character included in the program was not relatable or representative. Overall, participants stated that the program was useful in helping them to think about and articulate their healthcare values and priorities. The findings from the research will directly inform a small-scale pilot grant that will assess the acceptability, feasibility, and potential efficacy of a values solicitation and operationalization intervention for AAs with MCCs and caregivers.

